# Lentivirus Susceptibility in Iranian and German Sheep Assessed by Determination of *TMEM154* E35K

**DOI:** 10.3390/ani9090685

**Published:** 2019-09-15

**Authors:** Vahid Molaee, Vahid Otarod, Darab Abdollahi, Gesine Lühken

**Affiliations:** 1 Department of Animal Breeding and Genetics, Justus Liebig University Giessen, Ludwigstraße 21, 35390 Gießen, Germany; Vahid.Molaee@agrar.uni-giessen.de; 2 Quarantine and Biosafety Directorate General, Iran Veterinary Organization (IVO), Vali Asr Avenue, Seyd Jamaledin Asad Abadi Street, 6349 Tehran, Iran; votarod@hotmail.com; 3 Bureau of Animal Health and Disease Management, Iran Veterinary Organization (IVO), Vali Asr Avenue, Seyd Jamaledin Asad Abadi Street, 6349 Tehran, Iran; d.abdollahi@ivo.ir

**Keywords:** maedi-visna, small ruminant lentivirus, sheep, susceptibility, transmembrane protein, TMEM154

## Abstract

**Simple Summary:**

There are no data on the effect of the transmembrane protein 154 (TMEM154) E35K variants on susceptibility to small ruminant lentivirus (SRLV) infection in Iranian sheep breeds, and only limited data for German sheep flocks. This study aimed at investigating the association of *TMEM154* variants and SRLV infection status in Iranian and German sheep flocks and breeds. Three out of the four analyzed sheep flocks/breeds showed a significant association between *TMEM154* variants and SRLV prevalence. A complementary analysis was carried out based on regression analysis to test the relationship between frequency of the *TMEM154* E allele and SRLV prevalence in different flocks/breeds. Results showed that the *TMEM154* E allele frequencies could be useful for predicting genetic susceptibility to SRLV infection in a sheep flock or breed. Finally, the genetic susceptibility of different Iranian and German sheep breeds was compared based on the frequency of the *TMEM154* E allele.

**Abstract:**

Small ruminant lentiviruses (SRLVs) cause maedi-visna disease in sheep and are prevalent in Iran and Germany. The association of the transmembrane protein 154 (TMEM154) variants with SRLV infection has been previously identified by a genome-wide association (GWAS) approach and subsequent analyses, and validated in some US, German, and Turkish sheep flocks. We aimed at evaluating these findings for the first time in Iranian, and in some more German sheep flocks/breeds. Also, we aimed at comparing the SRLV susceptibility in Iranian and German sheep based on the frequency of the *TMEM154* E35 allele. About 800 blood samples were collected from 21 Iranian and German sheep flocks/breeds for different purposes: (1) The association of *TMEM154* E35K with SRLV infection status was tested in four sheep breeds and found to be significant in Kermani, Merinoland, and Brown Hair. (2) The usefulness of the *TMEM154* E35 frequency for predicting SRLV susceptibility was evaluated by regression analysis, combining data from this study and some already published data. Results showed a significant association between E35 frequency and SRLV prevalence. (3) SRLV susceptibility was compared based on E35 frequency in Iranian and German sheep. Altogether, findings of this study provide valuable information on SRLV susceptibility, using *TMEM154* E35, in Iranian and German sheep.

## 1. Introduction

Lentiviruses can cause maedi-visna (MV) disease in sheep and caprine arthritis encephalitis disease (CAE) in goats. Since these viruses infect both host species, they are generally called small ruminant lentiviruses (SRLV). Leading to progressive pneumonia, early culling, lower lamb weight, and lamb mortality of infected ewes, SRLVs cause high economic losses [1].

There is not any vaccine or cure for SRLV infection in sheep and goats. Eradication programs have already been established in many countries by culling infected animals and feeding kids with virus-free colostrum [2]. This strategy comes with costs because of cull replacements and the maintenance of SRLV-free animals [3]. A prerequisite for this eradication strategy is establishing a reliable tool for identifying infected animals [4]. Currently, there are several different laboratory techniques for the diagnosis of SRLV infection. These include serological methods, such as enzyme-linked immune assay (ELISA) and agar gel immunodiffusion (AGID), and polymerase chain reaction (PCR)-based techniques, such as nested and real-time PCR [2,5]. There is no gold standard in virology to diagnose SRLVs, and especially different ELISAs are still under debate. As reviewed by Herrmann-Hoesing [6], PCR tests compared to indirect ELISA tests have 92% to 95% positive concordance and 87% to 88% negative concordance. The genome of SRLVs contains two non-coding genes of long terminal repeats (LTR) and six coding genes encoding for structural proteins of *gag*, *pol,* and *env* and non-structural proteins of *tat*, *rev*, and *vif*. The *gag* gene includes matrix (MA), capsid (CA), and nucleocapsid (NC) domains, and the *env* gene contains surface (SU) and transmembrane (TM) proteins. A part of the *gag* gene (CA) and a part of the *env* gene (TM) are conserved among SRLVs, which make them suitable for designing immunodominant epitopes for serological purposes [7] and primers for both conventional and quantitative PCR [8]. Therefore, the major segments of *gag* (CA) and *env* (TM) in SRLVs could be targeted for screening SRLVs in sheep and goat flocks.

Another preventive intervention is to breed sheep for lower SRLV infection susceptibility [3]. There is much evidence that the susceptibility to SRLV infection varies among different sheep breeds [9,10]. Recently, various sheep genes, such as *MHC, CCR5, TLRs,* transmembrane protein 154 (*TMEM154*), *APOBEC3,* and *ZNF389*, were studied and associated with SRLV infection [3,11]. The association of *TMEM154* variants with SRLV infection has been identified by a genome-wide association (GWAS) approach and subsequent analysis, and validated in some North American sheep populations as well as recently in German and Turkish sheep flocks [12,13,14,15]. According to this, the susceptibility of sheep breeds to SRLV infection was assessed based on the frequency of *TMEM154* haplotypes [12,13,15] and alleles [14]. Twelve haplotypes were identified based on sequence variations in the *TMEM154* coding region. Haplotypes 2 and 3 (ancestral) carry a nucleotide (g) coding for glutamic acid (E) at position 35, whereas this nucleotide is substituted by a nucleotide (a) in haplotype 1 coding for lysine (K) [12]. In a study by Heaton et al. [16], using 50K single nucleotide polymorphism (SNP) chip data, the susceptibility of 74 globally distributed sheep breeds to SRLV infection was predicted based on the frequency of allele “c” which is in linkage disequilibrium (LD) with allele E at position 35 of *TMEM154* [16]. However, at present, the susceptibility status of certain sheep breeds cannot be predicted only based on the knowledge of the frequencies of *TMEM154* variants (alleles or haplotypes) or linked markers. In a German study, among the sheep breeds tested for association of *TMEM154* E/K with SRLV susceptibility, the results for Merinoland sheep and crossbreds of this breed indicated a possible deviation from the findings in other breeds, as a relatively high number of animals with the genotype KK were found to be serologically SRLV positive [14].

Iran is one of the main producers of small ruminants in the world. According to the large-scale surveys of the Statistical Center of Iran (SCI) in winter 2011, the population of small ruminants of Iran was composed of 50.2 million heads of sheep and 22.1 million heads of goats. The sheep population of Iran comprises 27 different sheep breeds, which were classified based on physical appearances such as size, shape, color, horn status, and tail type [17]. In Iran, SRLV infection was first diagnosed in the southwest of Iran (Khuzestan Province) using histopathological methods [18]. Later, many reports restricted to local areas of Iran, either using serological methods or PCR techniques, have shown a variable level (2.2% to 34.5%) of SRLV prevalence in sheep [19,20,21,22]. 

A prerequisite for the practical usage of a genetic marker is to validate the observed association with the trait of interest in other populations and countries. In the US studies [12,13] as well as a Turkish study [15], the association between *TMEM154* variants and SRLV infection status was evaluated based on frequencies of *TMEM154* haplotypes. However, we decided to select the single E allele at position 35 as a surrogate for the two most common highly-susceptible *TMEM154* haplotypes (i.e., “2” and “3”), and K35 as a surrogate for the most common *TMEM154* haplotype with reduced susceptibility (i.e., “1”). Together, these three haplotypes are present in about 98% of US sheep [12] and 86% of Turkish sheep [15]. Investigating the association of *TMEM154* E35K allele/genotype frequencies with SRLV susceptibility in Iranian sheep breeds was the main goal of the present study. A second aim was to validate previous observations in the German sheep population by including further sheep breeds and an additional flock of the Merinoland sheep breed from another region. Finally, we aimed at comparing SRLV susceptibility in different sheep breeds from Iran and Germany based on E or c allele frequencies. 

## 2. Materials and Methods 

### 2.1. Animals and Blood Collection 

For this study, three sets of samples from different sheep breeds of Iran (set 1) and Germany (sets 2 and 3) were collected.

The first set of blood samples (set 1, *n* = 365), originating from 30 flocks located in six provinces of Iran, were collected between 2015 and 2016. All sampled sheep were between four and seven years old and purebreds from the following sheep breeds: Makouee, Qezel, Moghani, Bakhtiari, Kaboudeh, Kermani, Balouchi, and Karakul (details on numbers of flocks, sheep per breed, and province are given in Table 1). There were no clinical signs of SRLV infection at the time of sampling and flocks were without any history of SRLV infection. No animal transfer happened between the sampled flocks. Therefore, all flocks were epidemiologically unrelated to each other. No other species than sheep were kept in each flock. 

The second set of samples (set 2, *n* = 127) was collected from four SRLV positive sheep flocks located in four provinces of Germany. Set 2 included samples from breeds Texel, Merinoland, Brown Hair, and Zackel (Table 1).

The third set of samples (set 3) included 302 samples from 11 pure sheep breeds of Germany which were collected during the years 2003–2018 for routine breeding requirements (e.g., parentage testing, prion protein genotyping) (Appendix A Table A1). As this set of samples was used to estimate *TMEM154* E/K allele frequencies within breeds, not more than three sheep originated from the same flock in order to minimize possible relationships between animals. There were no epidemiological data about SRLV infection status available for this set of samples. 

All blood samples (10 mL each) were drawn by puncture of the jugular vein and collected into ethylene-diamine-tetra-acetic acid (EDTA) tubes for DNA isolation. 

### 2.2. Ethics Statements

Blood samples were collected by veterinarians. Independent from this study, the original purpose of sampling was to test the MV status of sheep flocks (sample sets 1 and 2) or the scrapie resistance status of sheep (sample set 3) in order to decide on subsequent veterinary or breeding measures. According to the German Animal Welfare Act (released on 18th of May 2006, last changes on 17th of December 2018), regulations for animal protection, this origin of samples obviates the need for an explicit ethics committee approval. The Iranian Veterinary Organization (IVO) was directly responsible for gathering Iranian sheep samples as a part of routine examinations for care and control of animal diseases (permission released on 20th of December 2014, tracking number 93/22/70521). The ethics responsibility for the collection of Iranian sheep samples was in accordance with the legal requirements of that national authority. 

### 2.3. DNA Extraction

Genomic DNA was obtained from peripheral blood leukocytes by using a DNA extraction kit (MBST, Tehran, Iran) for Iranian sheep samples in accordance to the manufacturer’s protocol and for German sheep samples by a modified salting-out method [23]. 

### 2.4. Quantity and Quality Control of Extracted DNA

The concentration (quantity) and OD260/280 ratio (quality) of the extracted DNA were measured with the Nanodrop ND-1000 spectrophotometer (Thermo Fisher Scientific Inc., Waltham, MA, USA). Based on these results, the concentration of all DNA samples was standardized to the same value. Additionally, the integrity of the extracted DNA was determined by amplifying *SRY* and *AMLX* genes usually used for sex determination [24] (details on primers are given in Appendix A Table A2). The clear detection of PCR products displaying the male or female sex was a prerequisite for using a DNA sample in further analyses.

### 2.5. PCR Amplification for Detection of SRLVs in Iranian Sheep Samples (Set 1) 

**Figure A1:**
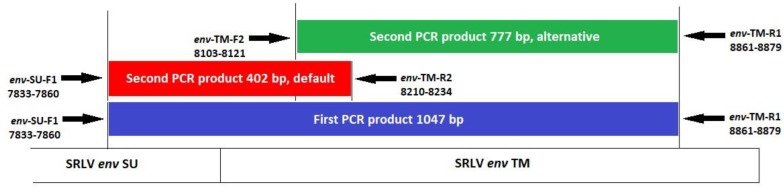
Primer localization in the *env* SU/TM region of small ruminant lentiviruses. Position of primers is according to SRLV strain K1514 (accession number: M60609). SU, surface protein; TM, Transmembrane protein.

Due to animal health requirements, serum samples of Iranian sheep breeds could not be shipped to Germany, where all laboratory analyses, except DNA extraction from Iranian samples, were carried out. Hence, SRLV infection status of Iranian sheep was determined by PCR test. For this purpose, a semi-nested PCR was designed corresponding to the *env* SU/TM fragment, by selecting the conserved regions with the highest sequence homology between different SRLV genotypes (A–C and E). Detailed information on primers is given in Table A2. To amplify a first PCR product with a size of ∼1 kb, the forward (*env*-SU-F1) and the reverse primer (*env*-TM-R1) were used. For the second PCR product (default PCR), an internal reverse primer (*env*-TM-R2) was used with the forward primer of the first PCR (*env*-SU-F1), providing a ∼0.4 kb fragment (Appendix A Figure A1, Table A2). The first PCR was carried out in a total volume of 15.0 µL, containing 150 ng template DNA, 1× Go Taq Flexi PCR buffer (Promega, Mannheim, Germany), 0.2 mM dNTPs, 1.5 mM MgCl_2_, 9 pmol of each primer (*env*-SU-F1 and *env*-TM-R1), and 0.6 units Go Taq-polymerase (Promega, Mannheim, Germany). The conditions for PCR were 94 °C for 1.5 min, followed by 35 cycles at 94 °C for 45 s, 55 °C for 1 min, 72 °C for 1 min, and a final extension at 72 °C for 5 min. The second PCR (default PCR) was carried out in a total volume of 50.0 µL, containing 3.0 µL of the first PCR product, 1× Go Taq Flexi PCR buffer (Promega, Mannheim, Germany), 0.2 mM dNTPs, 1.5 mM MgCl_2_, 30 pmol of each primer (*env*-SU-F1 and *env*-TM-R2), and 2 units Go Taq-polymerase. The conditions for the second PCR (default PCR) were 94 °C for 1.5 min, followed by 35 cycles at 94 °C for 20 s, 55 °C for 30 s, 72 °C for 45 s, and a final extension at 72 °C for 5 min. 

In order to validate the PCR results (default PCR), the second PCR was also carried out with an alternative primer pair, now using a new forward primer (*env*-TM-F2) together with the *env*-TM-R1 primer, resulting in a ∼0.8 kb fragment (Figure A1, Table A2). The second PCR (alternative) was carried out in a total volume of 25.0 µL, containing 1.5 µL of the first PCR product, 1× Go Taq Flexi PCR buffer (Promega, Mannheim, Germany), 0.2 mM dNTPs, 1.5 mM MgCl_2_, 12.5 pmol of each primer (*env*-TM-F2 and *env*-TM-R1), and 1 unit Go Taq-polymerase. The condition for the second PCR (alternative) was 94 °C for 1.5 min, followed by 40 cycles at 94 °C for 30 s, 58 °C for 45 s, 72 °C for 50 s, and a final extension at 72 °C for 10 min. 

For each amplification, a positive control (DNA from German sheep serologically diagnosed to be SRLV positive) [14] and a negative control (no DNA template) were run in parallel with all samples. All PCR products were analyzed by 1.7% agarose gel electrophoresis and visualized with ethidium bromide staining.

### 2.6. Serological Test for Detection of SRLVs in German Sheep Samples (Set 2)

A serological test with ELISA (IDEXX CAEV-MVV Total Ab ELISA, IDEXX GmbH, Ludwigsburg, Germany) as described previously [14] was carried out to determine the SRLV infection status in German sheep flocks (set 2). The used ELISA is an indirect ELISA designed to detect an immunogenic peptide of the transmembrane protein (*env* gene, TM) and the recombinant P28 protein which enters into the composition of the viral capsid (*gag* gene) (POURQUIER ELISA Maedi-Visna/CAEV Serum Verification, version P00302/07).

### 2.7. TMEM154 Genotyping

A total of 794 sheep samples from sets 1–3 were genotyped for *TMEM154* E35K by allele-specific PCR method. Details of the established method for genotyping of *TMEM154* E35K were described elsewhere [14].

### 2.8. Data Analysis

The SPSS program (version 25.0) for Windows (IBM SPSS Statistics, IBM Corp, Armonk, NY, USA) was used for statistical analyses. Differences in the distribution of genotype frequencies between sets of SRLV negative and positive samples were tested by chi-square or Fisher’s exact test (when expected values were < 5). The relative risk analyses were conducted based on the method of Altman [25].

Association analyses using chi-square/Fisher’s exact test were done only for those sheep flocks/breeds that complied with two conditions: (1) They had an SRLV prevalence between 10% and 90%, and (2) They included carriers of protective (KK) as well as of non-protective (EK or EE) genotypes based on a protocol by Clarke et al. [26].

For regression analysis, the average allele frequencies of the *TMEM154 E* allele in SRLV affected flocks of the same breed (or dominated by the same breed) were plotted against the average SRLV infection in the flocks of these breeds/breed mixes. This meta-analysis includes data generated in the present study (four sheep breeds from Iran (set 1, only positive flocks) and four sheep breeds from Germany (set 2), a previous German study (four sheep breeds/breed mixes) [14] and a previous US study (eight sheep breeds) [12].

For comparing SRLV susceptibility between different sheep breeds of Iran and Germany, in addition to E allele frequencies, c allele frequencies of SNP no. 5388531 (OVAR 17) located in the intronic region of *TMEM154* were extracted from 50K SNP chip data (International Sheep Genomics Consortium, ISGC, https://www.sheephapmap.org). Information on *TMEM154* E35 allele frequencies (combined frequencies of haplotypes 2 and 3) in three US sheep [12] were also used for comparison purposes.

## 3. Results

### 3.1. SRLV Infection Status of Iranian and German Sheep Flocks (Sets 1 and 2)

A proportion of 61 out of 365 (16.7%) Iranian sheep samples were determined to be SRLV positive by PCR test. It should be noted that changing primers and arrangement of the second PCR (alternative instead of default PCR) did not result in a different PCR status (positive or negative) of samples. In 10 out of 30 sampled Iranian sheep flocks (33%), at least one SRLV positive sheep was identified by PCR (Table 1). The SRLV prevalence ranged from 5% (flock no. 11) to 89% (flock no. 13) in single flocks. Samples with SRLV positive status were identified in the four sheep breeds Bakhtiari (all three sampled flocks), Kermani (two out of three flocks), Makouee (four out of nine flocks), and Qezel (one out of two flocks). 

In the German sample set (set 2), 90 out of 127 (71%) samples were tested SRLV positive by ELISA diagnosis. The SRLV prevalence in the German sheep breeds ranged from 48% (Brown Hair, flock no. 33) to 100% (Texel, flock no. 31). Details on SRLV infection status within Iranian and German flocks are given in Table 1.

### 3.2. TMEM154 Genotyping and Association Analyses (Chi-Square and Fisher’s Exact Test) 

All Iranian and German sheep samples were successfully genotyped for the amino acid substitution at position 35 of *TMEM154* (sets 1–3). 

Association analyses were limited to two sheep breeds from Iran (Kermani and Makouee) and two sheep breeds from Germany (Merinoland sheep and Brown Hair) (Table 2). A significant association (*p* < 0.05) between *TMEM154* genotypes at position 35 and SRLV status (positive vs. negative) was found in three of these analyzed sheep breeds, but not in Makouee from Iran. 

In the four analyzed flocks/breeds, the relative risk to be SRLV positive for sheep carrying one or two E alleles compared to those without E allele ranged from 0.48 to 20 (Table 2). In the Kermani sheep breed, the risk to be SRLV positive with E allele was almost half (0.48) compared to sheep with KK. In the German Brown Hair sheep breed, a noticeable high relative risk (20.0) was found for sheep with one or two copies of risk allele E compared to sheep without risk allele (KK). In this sheep breed, all sheep with the protective genotype (KK) were SRLV negative. 

*TMEM154* genotyping results of sheep flocks/breeds excluded from association analyses for different reasons (less than 10% or more than 90% SRLV positive sheep and/or non-adequate balance of genotype frequencies) are shown in Appendix A Table A3.

### 3.3. Regression Analysis Based on a Combination of Previously Published Data and Those of the Present Study

**Figure 1:**
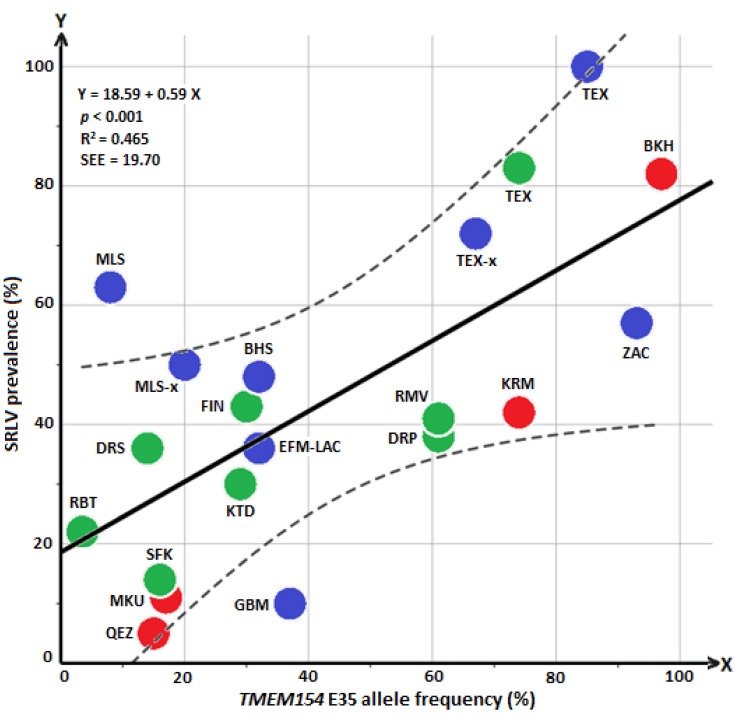
Relationship between *TMEM154* E allele frequency (%) and SRLV prevalence (%) in 20 different breeds/flocks from Iran, Germany, and the USA is shown by regression line (black). The dashed lines indicate 99% confidence interval. Sheep breeds/flocks of Iran, Germany, and the USA were labeled with red, blue and green solid colors, respectively. Breed names were abbreviated for Iran (Bakhtiari, BKH; Makouee, MKU; Kermani, KRM; Qezel, QEZ), Germany (present study: German Texel, TEX; Merinoland sheep, MLS; Brown Hair sheep, BHS; Zackel, ZAC; published by Molaee et al. [14]: purebred and crossbred German Texel sheep, TEX-x; German Blackheaded Mutton, GBM; purebred and crossbred Merinoland, MLS-x; East Friesian milk and Lacaune, EFM–LAC) and USA (published by Heaton et al. [12]: Dorset, DRS; Dorper, DRP; Finnsheep, FIN; Kathahdin, KTD; Rambouillet, RBT; Romanov, RMV; Suffolk, SFK; Texel, TEX). SEE, standard error of estimate.

A regression analysis was conducted with all available data from different sheep flocks/breeds of Iran, Germany, and data from North America. The regression line indicates a significant relationship (*p* < 0.001) between changes in the frequency of the allele E and changes in SRLV prevalence (Figure 1). The R-squared measure and standard error of estimate (SSE) were 0.465 and 19.70%, respectively. 

### 3.4. SRLV Susceptibility in Sheep Breeds of Iran and Germany Based on the Frequency of TMEM154 Alleles E and c

**Figure 2:**
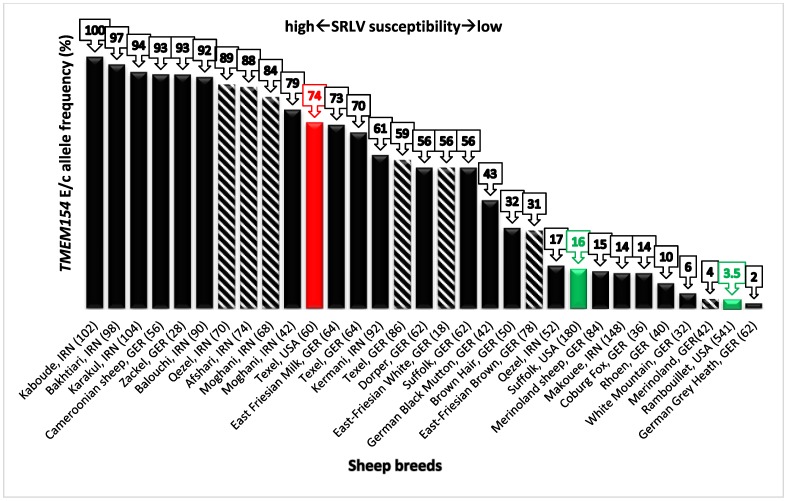
The degree of susceptibility to SRLV infection in nine sheep breeds from Iran (IRN) and fifteen sheep breeds from Germany (GER) is shown based on the frequency of the *TMEM154* E allele (solid bars, present study) or the c allele of SNP OVAR17-5388531 (shaded bars). The c allele is in linkage disequilibrium with the E allele. The c allele frequencies derived from 50K SNP chip data (International Sheep Genomics Consortium, ISGC). Colored solid bars indicate certain US sheep breeds with known SRLV susceptibility based on epidemiological data [12] (red = high susceptibility, green = low susceptibility).

Frequency of the E allele at position 35 of *TMEM154* found in different sheep breeds of Iran (*n* = 8) and Germany (*n* = 13) (sets 1–3), as well as the frequency of the intronic c allele (*n* = 7), are shown in Figure 2. For some sheep breeds, frequency information on both alleles (E and c) was available (*n* = 4). Information on E allele frequency (haplotypes 2 and 3) of three US sheep breeds with a known SRLV susceptibility status [12] was also included in the diagram as a criterion for comparing SRLV susceptibility of different sheep breeds. E/c allele frequencies ranged from 2% (German Grey Heath, Germany) to 100% (Kaboudeh, Iran) with a median of 56% and interquartile range from 15% (comparable with E allele frequencies of the US Suffolk) to 88% (comparable with E allele frequencies of the US Texel). In most of the analyzed Iranian sheep breeds, the observed E/c allele frequencies were relatively high. In five of them, the E/c allele frequencies were higher than or equal to the third quartile value (≥88%), in three of them, the E/c allele frequencies were lower than the first quartile value. In the breed Qezel, E and c allele frequencies were not congruent. In the German data set, E/c allele frequencies mostly ranged between quartile 1 and quartile 3 values (15–88%). In five out of the 15 analyzed breeds, E/c allele frequencies were lower than first quartile value (≤15%), whereas, in two breeds, E allele frequencies were higher than the third quartile value. 

## 4. Discussion

This study is the first to assess the usefulness of an amino acid substitution (E/K) in *TMEM154* for selection against SRLV susceptibility in sheep breeds of Iran. Moreover, it enlarges existing data for the German sheep population. SRLV positive samples were found in flocks of eight different sheep breeds, and in four of them, the association of *TMEM154* E35K with SRLV status of sheep was tested applying the chi-square/Fisher’s exact test. A significant association was found in flocks of the breeds Kermani, Merinoland and Brown Hair. Data from flocks of the other four breeds were not suitable for this method of association testing. To provide adequate power in testing for statistical significance it is necessary to find flocks with a “moderate” level of infection (e.g., not all or most sheep should be positive or negative) and a balanced ratio of genetically susceptible and resistant sheep. This is why in a study by Leymaster et al. [13] ewes which were infected and had *TMEM154* diplotype “1,3” were mated with “1,3” rams so that lambs with *TMEM154* diplotypes “1,1”, “1,3” and “3,3” were produced in a 1:2:1 ratio in an environment with maximum pathogen exposure. The outcome was nearly 100% infected lambs with either one or two copies of haplotype “3” (EK/EE) and only 10% infected with haplotype “1,1” (KK) after five years. Of course, such optimal conditions are hard to find in field studies. For example, in the present study association testing for the Qezel flock with only 5% infected sheep was prevented because the SRLV prevalence was too low. We also had to withhold the Bakhtiari, German Texel and Zackel flocks/breeds from conventional association analyses because of the absence of the KK genotype. However, in these three breeds, a high SRLV prevalence came along with a high E allele frequency. Accordingly, a low E frequency was related to a low SRLV prevalence in Qezel. This positive correlation between E allele frequency and SRLV prevalence could be demonstrated by regression analysis, including data of sheep flocks/breeds from Iran, Germany, and the USA (Figure 1). Regarding the regression line, an SRLV prevalence of about 30% is expected in infected flocks with an E frequency of 20%, while an SRLV prevalence of about 60% is expected in sheep flocks with an E frequency of 70% (Figure 1). Regression analysis was used for two reasons: (1) independently from any prerequisite for carrying out a conventional association analysis, the regression analysis was helpful to visualize the relationship between “SRLV prevalence” and “E allele frequency” in all eight positive breeds of this study; (2) a general agreement between frequency of risk allele/genotypes of breeds and SRLV infection status was already observed by Heaton et al. [12]. Thus, we aimed to examine these relationships with a combined data set from sheep flocks of different countries (Iran, Germany, and the US), using regression analysis. Regression analysis showed a moderate R-square (R^2^ > 0.6, strong accuracies; 0.4 < R^2^ < 0.6, moderate accuracies). For instance, an SRLV prevalence of about 62% would be predicted for flocks/breeds with an E allele frequency of about 75% (Figure 1; “KRM” and “TEX”), with a range from 42 to 82% (SEE ± 20%). With regard to prediction accuracy, a moderate value is common for most of the traits which are controlled by multiple genes, including susceptibility to disease [3]. Other factors such as effect of the different SRLV subgroups [27], other possible gene regions [3,11], and management also may prevent a precise prediction of the susceptibility to SRLV infection based on the *TMEM154* E allele frequency. 

The relative risk levels estimated for sheep carrying one or two E alleles in the four analyzed flocks/breeds in this study (Kermani, RR = 0.48 to Makouee, RR = 2.18) were, with one exception (Brown Hair, RR = 20), comparable with previous studies in German [14] and US sheep flocks [12]. White and Knowles [3] explained that a ∼2.5-fold relative risk for *TMEM154* haplotypes 2 and 3 was quite large for a single gene. The estimated relative risk for Merinoland sheep carrying one or two copies of the E allele was in the same range (95% Cl = 1.36 to 2.34) as previously estimated for this breed with another set of samples from another German region [14]. For the analyzed flock of Brown Hair sheep, an outstanding high risk (RR = 20) for sheep carrying one or two copies of the E allele was obtained with a high confidence interval because of zero denominators (absence of positive sheep with KK genotype). 

In this study, SRLV susceptibility of sheep breeds of Iran and Germany was compared based on frequencies of the *TMEM154* E and/or c allele (the latter located in an intronic region of *TMEM154*). For the breeds Moghani, Texel, Merinoland, and Qezel, frequency information was available for both the E and the c allele. The frequencies of E and c alleles matched for all these breeds but not for Qezel (17 vs. 89%). Heaton and colleagues [12,16] showed a strong LD (*p*-value = 3.19 × 10^−9^, r^2^ = 0.98) between the causative allele (E) and the c allele in an intron of *TMEM154* in US sheep. The question is, in the light of the possible age of the *TMEM154* variants, which most likely occurred before modern breed separation, if the used SNP marker from the ovine SNP array is a good predictor for the close coding variant. In this study, with the example of the Iranian Qezel breed, we were able to show that data on the frequency of the c allele, which are available for a high number of international sheep breeds, can result in a wrong assessment of the SRLV susceptibility of a breed. Hence, the knowledge of the *TMEM154* E35 frequency is a crucial factor for the assessment of SRLV susceptibility of a single breed. However, another explanation may be related to the relatively low sample size for this breed in the present study and also for the generation of the ovine SNP chip data (26 and 35 sheep, respectively), maybe leading to an inaccurate estimation of the E and c allele frequencies for this breed. Although a low E allele frequency is consistent with the low SRLV prevalence (5%) in the Qezel flock, further sampling from additional flocks is necessary to provide a more accurate estimation of the SRLV susceptibility of this breed. 

In Germany, the three sheep breeds Texel, East Friesian Milk, and Cameroon are historically known to be highly susceptible to SRLV infection. In contrast, the three sheep breeds German Grey Heath, Merinoland, and Suffolk are commonly not affected by this disease. The *TMEM154* E allele frequencies of these breeds are consistent with these observations. Additionally, in the Merinoland sheep breed, the relatively low E allele frequency seems to reflect the “general” low susceptibility of this breed [28]. For this reason, it was quite surprising to repeatedly find a high number of KK sheep (55% in this study; 44% in a previous study [14]) to be serologically SRLV positive in flocks of the Merinoland sheep breed. A follow-up GWAS study could be useful for detecting potential additional variants/markers related to SRLV infection status in this breed. 

The SRLV infection status observed in Iranian sheep flocks (Table 1) was consistent with earlier studies. Nevertheless, we did not find any positive samples in the province of Khorasan Razavi that belonged to the northeast of Iran (Table 1, flock nos. 23–30), whereas serological studies have shown about 20% [22] and 35% [20] seropositivity in sheep of that region. It could be possible that our sampling missed infected flocks in this province. Furthermore, unknown mutations in the provirus sequences could have created primer mismatches and consequently prevented PCR amplification. However, this will only be an explanation if other SRLV strains are present in this region. There has been no report of any SRLV infection in the Fars Province, and we also did not find positive samples in this area. In this study, screening for the presence of SRLV infection was done for the first time from the provinces Western Azarbaijan and Ardebil. SRLV positive samples were found in the province of Western Azarbaijan, which is at the border of Turkey. In a follow-up study, SRLV sequences from Iran should be compared with those already observed in Turkey [29] in order to gain a deeper insight into the variability of SRLVs of the entire region. This could also help to develop more precise tools for diagnosis and prevention of SRLV infection in sheep.

### 4.1. Limitation of the Study

In other studies, it has been shown that rare E35-containing haplotypes also can confer recessively a lower susceptibility to SRLV (i.e., haplotype “4” together with haplotype “16” according to the study of Yaman et al. [15]). Therefore, the findings of the present study should not only be verified with more samples from additional flocks and breeds, but also with *TMEM154* haplotype data.

## 5. Conclusions

In this study, a significant association was found between *TMEM154* E35K variants and SRLV infection status in three out of four analyzed sheep breeds. Although our results showed a significant association in the flock of Merinoland sheep, a high number of KK sheep (carrier of the protective genotype) were SRLV positive. A follow-up GWAS study could be useful for detecting potential additional variants/markers related to SRLV infection status in Merinoland sheep.

An additional point of this study was testing the usefulness of the frequency of the *TMEM154* E allele for prediction of SRLV susceptibility in sheep flocks or breeds. Regression analysis showed that the SRLV susceptibility can be predicted based on the frequency of allele E with a variation of about plus/minus 20%.

Additionally, we found, for the first time and by a PCR-based test, SRLV positive sheep flocks in the Iranian province of Western Azarbaijan. Characterization of SRLVs in this province (located on Iran's border with Turkey), as well as other regions of Iran, will provide insight into the variability of SRLVs of the entire region.

## Figures and Tables

**Table 1:** Small ruminant lentivirus (SRLV) infection status in sampled sheep flocks of Iran and Germany.

Flock No.	Country	Province	Breed	Sampled Sheep (*n*)		Positive ^1^
1	Iran	Western Azarbaijan	Makouee	9		1 (11%)
2	Iran	Western Azarbaijan	Makouee		7	0 (0%)
3	Iran	Western Azarbaijan	Makouee		5	0 (0%)
4	Iran	Western Azarbaijan	Makouee		7	1 (14%)
5	Iran	Western Azarbaijan	Makouee		9	1 (11%)
6	Iran	Western Azarbaijan	Makouee		10	0 (0%)
7	Iran	Western Azarbaijan	Makouee		10	1 (10%)
8	Iran	Western Azarbaijan	Makouee		4	0 (0%)
9	Iran	Western Azarbaijan	Makouee		13	0 (0%)
10	Iran	Western Azarbaijan	Qezel		6	0 (0%)
11	Iran	Western Azarbaijan	Qezel		20	1 (5%)
12	Iran	Ardebil	Moghani		21	0 (0%)
13	Iran	Chaharmahal-Va-Bakhtiari	Bakhtiari		18	16 (89%)
14	Iran	Chaharmahal-Va-Bakhtiari	Bakhtiari		15	10 (67%)
15	Iran	Chaharmahal-Va-Bakhtiari	Bakhtiari		17	15 (88%)
16	Iran	Fars	Kaboudeh		10	0 (0%)
17	Iran	Fars	Kaboudeh		21	0 (0%)
18	Iran	Fars	Kaboudeh		12	0 (0%)
19	Iran	Fars	Kaboudeh		8	0 (0%)
20	Iran	Kerman	Kermani		14	3 (21%)
21	Iran	Kerman	Kermani		22	12 (55%)
22	Iran	Kerman	Kermani		10	0 (0%)
23	Iran	Khorasan Razavi	Balouchi		10	0 (0%)
24	Iran	Khorasan Razavi	Balouchi		2	0 (0%)
25	Iran	Khorasan Razavi	Balouchi		10	0 (0%)
26	Iran	Khorasan Razavi	Balouchi		23	0 (0%)
27	Iran	Khorasan Razavi	Karakul		12	0 (0%)
28	Iran	Khorasan Razavi	Karakul		11	0 (0%)
29	Iran	Khorasan Razavi	Karakul		9	0 (0%)
30	Iran	Khorasan Razavi	Karakul		20	0 (0%)
31	Germany	Schleswig-Holstein	Texel		39	39 (100%)
32	Germany	Bayern	Merinoland		49	31 (63%)
33	Germany	Nordrhein-Westfalen	Brown Hair		25	12 (48%)
34	Germany	Baden-Württemberg	Zackel		14	8 (57%)

^1^ SRLV positive samples in Iranian and German flocks were detected by PCR test (*env-*SU/TM) or serology (ELISA technique), respectively.

**Table 2:** *TMEM154* E35K genotype frequencies in four SRLV positive sheep breeds of Iran (IRN) and Germany (GER) with results from chi-square/Fisher’s exact test and relative risk (RR) analyses.

Breed Subset	MV Status	*TMEM154* Genotype Frequencies			*p* Value	RR	95% Cl	*p* Value
(*n* Sheep)	(*n* Sheep)	KK	EK	EE				
Makouee, IRN (35)	neg (31)	0.71 (22)	0.29 (9)	0.00 (0)	0.118	2.18	0.35 to 13.53	0.402
	pos (4)	0.50 (2)	0.25 (1)	0.25 (1)				
Kermani, IRN (36)	neg (21)	0.09 (2)	0.24 (5)	0.67 (14)	0.047	0.48	0.24 to 0.96	0.038
	pos (15)	0.33 (5)	0.00 (0)	0.67 (10)				
Merinoland, GER (49)	neg (18)	1.00 (18)	0.00 (0)	0.00 (0)	0.0197	1.782	1.359 to 2.337	<0001
	pos (31)	0.74 (23)	0.26 (8)	0.00 (0)				
Brown Hair, GER (25)	neg (13)	0.85 (11)	0.15 (2)	0.00 (0)	0.00002	20	1.313 to 304.494	0.0311
	pos (12)	0.00 (0)	0.83 (10)	0.17 (2)				

## Appendix A

**Table A1:** Samples collected from German breeding flocks (set 3).

Breed No.	Breed	Sheep (*n*)
1	German Grey Heath	31
2	White Moutain	16
3	Rhoen	20
4	Coburg Fox	18
5	Merinoland	42
6	German Black Mutton	21
7	Dorper	31
8	Suffolk	31
9	Texel	32
10	East Friesian Milk	32
11	Cameroonian sheep	28
Total		302

**Table A2:** Details on PCR amplifications done in this study.

Gene	Primer Sequences (5′ → 3′)	Purpose of PCR	Product Size	Reference
*TMEM154*	CCACAGGAGAGGAGRACACA (forward) GGGCACGTCTCCTGACAGTTT (reverse, FAM-labeled, matching with K allele) GGCACGTCTCCTGACAGTTC (reverse, HEX-labeled, matching with E allele)	genotyping *TMEM154* E35K	40/41 bp	[14]
*TMEM154*	GCTAGACACTGCCAAGCTTC (forward) TGTCACTGAAACAAGTCATCACT (reverse)	sequencing *TMEM154* region around E35K	788 bp	[14]
*env* Surf-Trans	TAATAARRGTRAGAGCTTACACATATGG (*env*-SU-F1) CCTGACAGTCCACCCTTTC (env-TM-R1)	diagnosis of SRLV infection, first PCR	1047 bp	this study
*env* Surf-Trans	TAATAARRGTRAGAGCTTACACATATGG (*env*-SU-F1) TTACACAGWAGTGTTGATAATGCCA (*env*-TM-R2)	diagnosis of SRLV infection, second PCR (default)	402 bp	this study
*env* Surf-Trans	ATGCCATGGTACAGCATGT (env-TM-F2) CCTGACAGTCCACCCTTTC (*env*-TM-R1)	diagnosis of SRLV infection, second PCR (alternative)	777 bp	this study
*AMELX* (*Capra hircus*)	CAGTAGCTCCAGCTCCAGCT (*Aml*.X-F1) GTGCATCCCTTCATTGGC (*Aml*.X-R1)	assessment of DNA quality	300 bp	[24]
*SRY* (*Capra hircus*)	ATGAATAGAACGGTGCAATCG (*Sry*-F) GAAGAGGTTTTCCCAAAGGC (*Sry*-R)	assessment of DNA quality	116 bp	[24]

**Table A3:** Genotyping results of *TMEM154* E35K variants in four positive sheep flocks of Iran (IRN) and Germany (GER).

Breed Subset	MV Status	*TMEM154* Genotype Frequencies		
(*n* Sheep)	(*n* Sheep)	KK	EK	EE
Bakhtiari, IRN (50)	neg (9)	0.00 (0)	0.11 (1)	0.89 (8)
	pos (41)	0.00 (0)	0.05 (2)	0.95 (39)
Qezel, IRN (20)	neg (19)	0.74 (14)	0.21 (4)	0.05 (1)
	pos (1)	1.00 (1)	0.00 (0)	0.00 (0)
Texel, GER (39)	neg (0)	0.00 (0)	0.00 (0)	0.00 (0)
	pos (39)	0.00 (0)	0.31 (12)	0.69 (27)
Zackel, GER (14)	neg (6)	0.00 (0)	0.17 (1)	0.83 (5)
	pos (8)	0.00 (0)	0.12 (1)	0.88 (7)
